# Cartilage organoids and osteoarthritis research: a narrative review

**DOI:** 10.3389/fbioe.2023.1278692

**Published:** 2023-11-09

**Authors:** Daofu Zeng, Yeping Chen, Zhidong Liao, Guizheng Wei, Xiajie Huang, Rongyuan Liang, William W. Lu, Dan Yi, Yan Chen

**Affiliations:** ^1^ Collaborative Innovation Centre of Regenerative Medicine and Medical BioResource Development and Application Co-Constructed by the Province and Ministry, Guangxi Medical University, Nanning, Guangxi, China; ^2^ Department of Bone and Joint Surgery, The First Affiliated Hospital of Guangxi Medical University, Nanning, Guangxi, China; ^3^ Department of Orthopedics and Traumatology, The University of Hong Kong, Pokfulam, Hong Kong SAR, China; ^4^ Research Center for Computer-Aided Drug Discovery, Shenzhen Institute of Advanced Technology, Chinese Academy of Sciences, Shenzhen, China; ^5^ Faculty of Pharmaceutical Sciences, Shenzhen Institute of Advanced Technology, Chinese Academy of Sciences, Shenzhen, China

**Keywords:** organoids, cartilage organoids, osteoarthritis, disease model, regeneration and repair

## Abstract

Osteoarthritis (OA) is one of the most common degenerative joint diseases, significantly impacting individuals and society. With the acceleration of global aging, the incidence of OA is increasing. The pathogenesis of osteoarthritis is not fully understood, and there is no effective way to alleviate the progression of osteoarthritis. Therefore, it is necessary to develop new disease models and seek new treatments for OA. Cartilage organoids are three-dimensional tissue masses that can simulate organ structure and physiological function and play an important role in disease modeling, drug screening, and regenerative medicine. This review will briefly analyze the research progress of OA, focusing on the construction and current development of cartilage organoids, and then describe the application of cartilage organoids in OA modeling, drug screening, and regeneration and repair of cartilage and bone defects. Finally, some challenges and prospects in the development of cartilaginous organoids are discussed.

## Introduction

**FIGURE 1:**
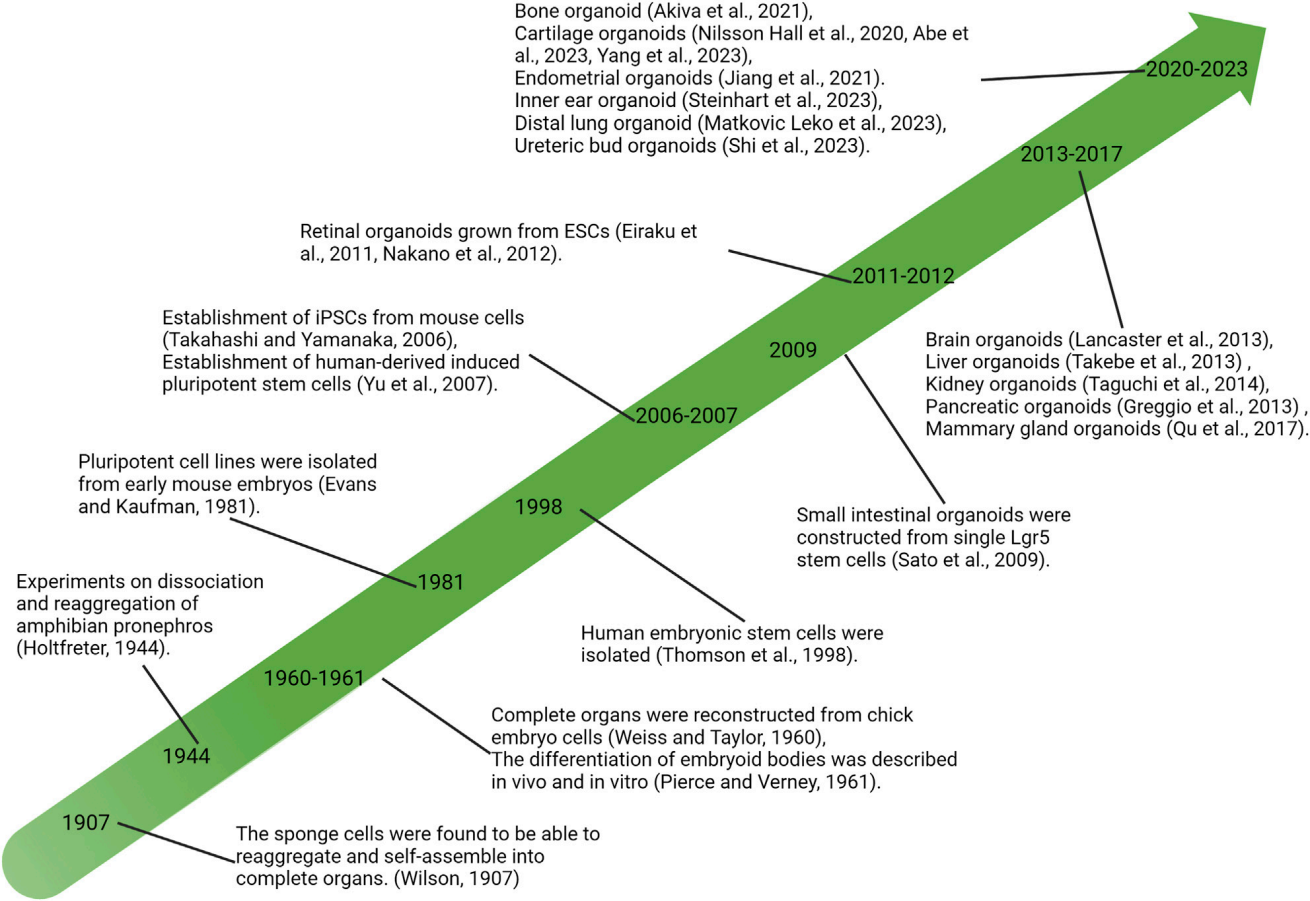
Significant research advances in history related to organoid development. PSCs, pluripotent stem cells; iPSC, induced pluripotent stem cells; ESCs, embryonic stem cells.

Lasting nearly a century of exploring developmental biology and cell biology, the self-organizing ability of vertebrate cells, i.e., the cells can reassemble and reconstruct the original structure of organs after complete separation, has been revealed and demonstrated (Lancaster and Knoblich, 2014). In the last century, *in vitro* experiments on tissue cells were extensively developed, and isolated sponge cells were first found to be able to reaggregate and self-organize into intact sponges in 1907 (Wilson, 1907), followed by the reconstruction of entire organs from chicken embryo cells (Weiss and Taylor, 1960). These discoveries provided a new understanding of cells’ self-organizing ability and further promoted the study of cell dissociation and reaggregation. Meanwhile, the discovery and research of stem cells are developing rapidly. In 1981, pluripotent stem cells (PSCs) were isolated and established from mice (Evans and Kaufman, 1981), and subsequently, human-derived embryonic stem cells (hESCs) were successfully isolated (Thomson et al., 1998; Yu et al., 2007). Since then, a large number of research efforts have led to a growing understanding of the functional behavior of stem cells and have begun to intervene in the fate of stem cells or progenitor cells at the molecular level, such as controlling specific differentiation directions (Holtfreter, 1944; Pierce and Verney, 1961). The development of stem cell biology and the understanding of the ability of cells to self-organize have contributed to the development of organ reconstruction research using stem or progenitor cells *in vitro*, resulting in the derivation of cartilaginous organoids (Figure 1).

Under appropriate culture conditions, by providing a corresponding three-dimensional culture environment, stem cells form a structure similar to the related tissues and organs, and this culture is called an organoid. Organoids originate from pluripotent or adult stem cells, which are capable of developing into a variety of cell types and, through spontaneous self-organization, form organ structures similar to those *in vivo* and have at least some of the functions of the corresponding organs (Rossi et al., 2018; He et al., 2020). Developing organoids is one of the essential achievements in stem cell research and marks a new stage in stem cell culture. The development of organoids has provided innovative means for the development of *in vitro* biological models and significantly promoted the evolution of developmental biology and stem cell biology. Organoids have a wide range of applications in basic biology, disease modeling, drug discovery, and regenerative medicine (Clevers, 2016).

Osteoarthritis (OA) is the most common disabling joint disorder and is associated with a high economic burden (Manivong et al., 2023). With the acceleration of global aging, the incidence of OA is increasing. Thus, seeking more promising methods for the prevention and treatment of OA is urgent. Currently, most studies on OA are based on animal models and cell cultures. However, due to the structural and physiological differences between species as well as the two-dimensional limitations, the actual situation of human homeostasis cannot be duplicated. Cartilage organoids are a new organoid model derived from organoid development (Kretzschmar and Clevers, 2016). Cartilage organoids have great potential in tissue development, disease modeling, and drug screening.

This review will briefly summarize the research progress of OA, focusing on the construction and current development status of cartilage organoids, and then describe the application of cartilage organoids in the OA modeling, drug screening, and regeneration and repair of bone and cartilage defect. Finally, some of the challenges and prospects in the development of cartilage organoids will be discussed.

## Osteoarthritis

The occurrence of OA is related to many factors, such as age, obesity, mechanical injuries, and genetic factors. The pathological changes of OA include cartilage destruction, synovial inflammation, subchondral bone sclerosis, and osteophyte formation. OA involves lesions of the whole joint structure, but articular cartilage damage is usually considered the central pathological feature. A complete joint structure ensures the normal function of the joint and provides appropriate resistance to stress (Martel-Pelletier et al., 2016). However, the structure and composition of articular cartilage will change during the development of OA. Mechanical damage or inflammation can disrupt chondrocytes’ homeostasis, leading to OA’s development and progression (Pritzker et al., 2006). The pathogenesis of OA is not yet fully clear. Disorders of decomposition and anabolism of chondrocytes are important factors in the occurrence and development of OA. Abnormal increases in chondrocyte catabolism, such as upregulated expression of cartilage matrix metalloproteinases (MMPs) and aggregate enzymes (ADAMTs), mediate the degradation of the cartilage matrix, leading to the occurrence of OA (Mueller and Tuan, 2011; Wang et al., 2013). In addition, the release of cartilage degradation products into the joints further promotes the inflammatory response and accelerates the development of OA (Mueller and Tuan, 2011; Wang et al., 2013).

Although OA has been studied for decades, the exact mechanisms leading to the abnormal expression of matrix-degrading enzymes have yet to be defined. Thanks to studies on animal experiments and cell models *in vitro*, it has been found that changes in a variety of signaling pathways may be involved in the occurrence of OA pathological state, such as the Wnt/β-catenin (Lietman et al., 2018), NF-kB (Lepetsos et al., 2019), AMPK (Zheng et al., 2021), BMP (Jaswal et al., 2023) signaling pathway. A deeper understanding of the interaction between these signals will provide new potential targets for the intervention and treatment of OA, and some compounds targeting the related signaling pathways may also become potential drugs for the treatment of OA (Ashruf and Ansari, 2022).

Most of the current OA treatments focus on relieving pain to improve quality of life. Clinically, non-drug treatments for OA mainly include physical therapy, weight loss, and active exercise. Non-steroidal anti-inflammatory drugs, glucocorticoids, and opioids such as morphine are used to relieve pain symptoms of OA but cannot stop the disease process (Arden et al., 2021). Damaged articular cartilage has a poor capacity for self-repair and there is so far no efficient curative treatment available for OA (Nazempour and Van Wie, 2016). Biological therapy has become a new research strategy for the treatment of OA, which is favored by a large number of researchers. For example, platelet-rich plasma (PRP) injection into the joint cavity can improve the clinical symptoms of patients (Sax et al., 2022). Furthermore, mesenchymal stem cell therapy has also shown a positive effect in the treatment of OA, which can relieve pain and improve joint function to a certain extent after the injection of stem cells (Pers et al., 2016; Harrell et al., 2019). However, the above biological therapy still faces some problems. First, pain is relieved after the treatment, but the pathological changes of OA are still not improved. Second, the survival rate of injected stem cells in the joint cavity is low, which cannot achieve sustained therapeutic effects. Last, more research is needed on the dose, cell type, and duration of intervention of biologically based therapies. Therefore, we still need to explore better models to study OA mechanisms and seek new therapeutic strategies.

## Definition and construction of cartilage organoids

In recent decades, research on organoids has developed rapidly, and remarkable achievements have been made in the small intestine (Sato et al., 2009; Sugimoto et al., 2018), brain (Lancaster et al., 2013; Mansour et al., 2018), liver (Coll et al., 2018), kidney (Takasato et al., 2014; Shi et al., 2023), lung (Matkovic Leko et al., 2023), and retina (Eiraku et al., 2011; Nakano et al., 2012; Völkner et al., 2016). The review by Fatehullah et al. summarizes other research progress on organoids (Fatehullah et al., 2016). However, the research on organoids in the skeletal system, particularly cartilage tissue, is still limited. Cartilage organoids are an extension of organoid development. They are formed by differentiation of embryonic stem cells (ESCs), induced pluripotent stem cells (iPSCs), or adult stem cells (ASCs) with self-renewal and self-organization ability and have the basic spatial structure and partial physiological functions of cartilage tissue (GBD, 2017 Disease and Injury Incidence and Prevalence Collaborators et al., 2018; Lin et al., 2023). Cartilage organoids have the basic spatial structure and partial physiological functions of cartilage tissue and have important potential value in the research and treatment of articular cartilage diseases.

**FIGURE 2:**
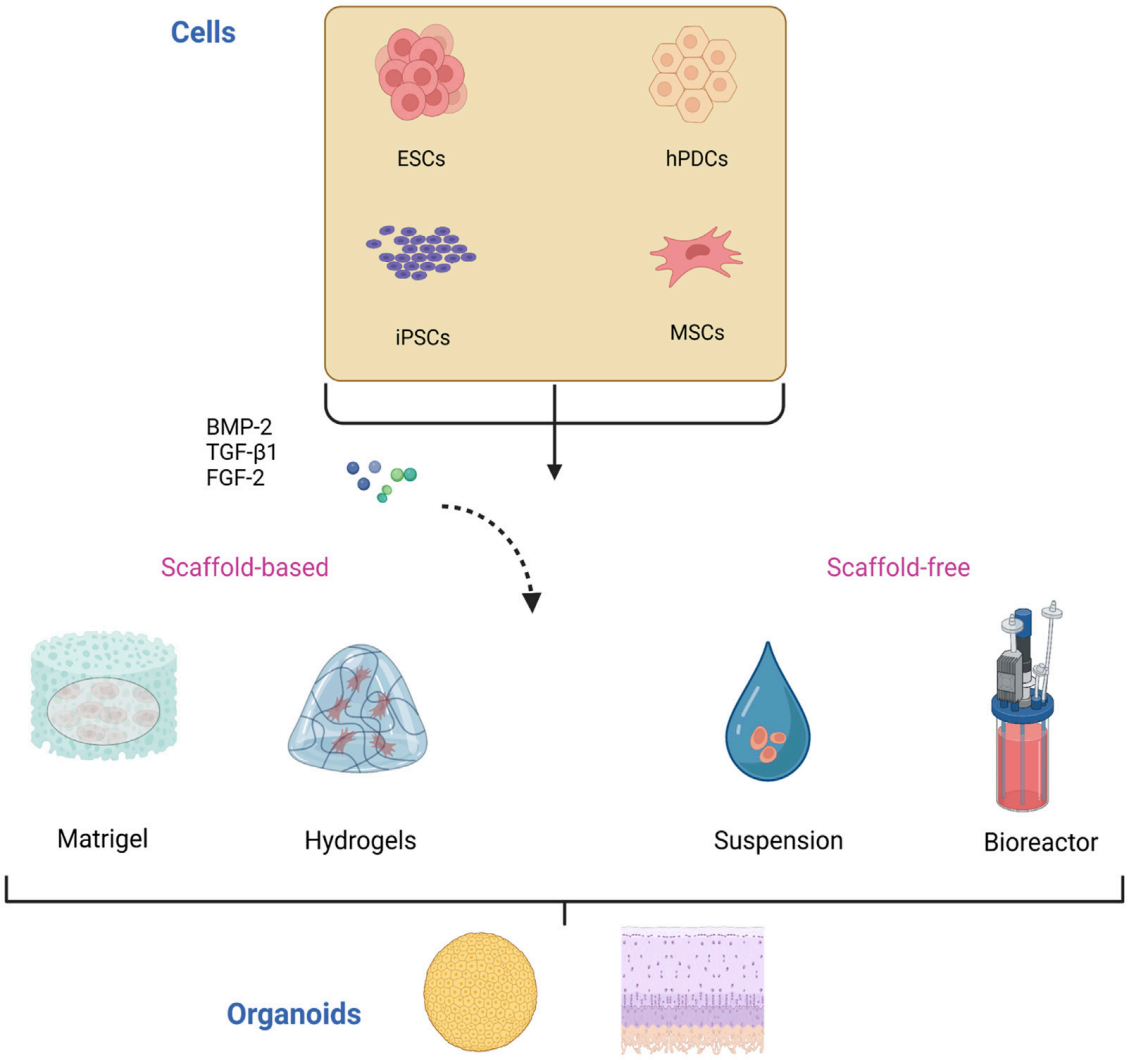
Construction of cartilage organoids. The cells used in the culture of cartilage organoids are mainly stem cells including ESCs, hPDCs, iPSCs, and MSCs. These cells are induced and differentiated by various differentiation and growth factors, including BMP-2, TGF-β1, and FGF-2, and then self-assemble into organoids in scaffold-based or scaffold-free culture. ESCs, embryonic stem cells; iPSCs, induced pluripotent stem cells; hPDCs, human periosteal-derived cells; MSCs, mesenchymal stem cells. BMP-2, bone morphogenetic protein 2; TGF-β1, transforming growth factor β1; FGF-2, fibroblast growth factor 2.

Several aspects need to be clarified for the construction of cartilage organoids (Figure 2). First, the original cell of the organoids, which is the main component of organoids establishment and plays a vital role in the physiological characteristics of organoids, should be determined. Second, it is necessary to construct a proper culture environment, select appropriate culture materials and biomaterials, and simulate the natural culturing environment to promote cell growth and differentiation. In addition, different culture methods affect the structure formation of organoids.

## Cell origin of cartilage organoids

The culture and proliferation of initiating cells is the first step in constructing organoids. The primary source of cells for cartilaginous organoids is from isolated and cultured stem cells, i.e., ESCs, iPSCs, mesenchymal stem cells (MSCs), human periosteal-derived cells (hPDCs), and other stem cells or pluripotent cell lines, as well as chondrocytes of autologous digestive origin.

### Embryonic stem cell

ESCs are totipotent cells derived from early mammalian embryos or primitive gonads, which have the characteristics of unlimited proliferation and multi-directional differentiation *in vitro*. The first isolation of ESCs was derived from mouse tissues. Evans and Kaufman first isolated embryonic stem cell lines from mouse embryos in 1981 and carried out preliminary culture and identification (Evans and Kaufman, 1981). Thomson et al. successfully isolated ESCs from human tissues in 1998 (Thomson et al., 1998). ESCs are pluripotent and capable of developing into various tissues in the three germ layers (ectoderm, mesoderm, and endoderm) through cell proliferation and induced differentiation. Cultured *in vitro*, ESCs can differentiate into functional hepatocytes and express hepatocyte markers such as tyrosine aminotransferase and secreted albumin (Cai et al., 2007). ESCs can be induced to differentiate into cardiomyocytes by successively adding activin A and bone morphogenetic protein 4 (BMP4) (Laflamme et al., 2007). Furthermore, ESCs can also differentiate into hematopoietic cells (Matsumoto et al., 2009), nerve cells (Reubinoff et al., 2001), alveolar epithelial cells (Wang et al., 2007), chondrocytes (Wang T. et al., 2019), and other tissue cells. The unlimited proliferation and differentiation pluripotency of ESCs have been used to construct various organs, such as liver organoids (Wang S. et al., 2019) and reproductive system organoids (Jiang et al., 2021). Correspondingly, ESCs have great potential in the study of cartilage organoids. Lauren et al. differentiated ESCs into neural crest stem cells and then further differentiated and self-assembled into craniofacial cartilage organoids (Foltz et al., 2021). Mass spectrometry analysis showed that cartilage organoids highly expressed cartilage marker proteins, providing new insights for studying craniofacial cartilage development and cell signaling (Foltz et al., 2021). However, the ethical consideration over separating ESCs has limited its application.

### Induced pluripotent stem cells

Using the reprogramming technique, differentiated cells can be restored to pluripotency, which is called induced pluripotent cells (Takahashi and Yamanaka, 2006). iPSCs were first induced in 2006 by Takahashi and Yamanaka, who restored the pluripotency of these cells by adding transcription factors during fibroblast culture, and they exhibited the morphology and characteristics of ESCs (Takahashi and Yamanaka, 2006). On the one hand, the successful creation of iPSCs dramatically solves the ethical concern ESCs face (Takebe et al., 2013; Taguchi et al., 2014). On the other hand, iPSCs can be induced from autologous mature somatic cells, avoiding the immune rejection of allogeneic transplantation (Okita et al., 2011). iPSCs have the characteristics of stem cells and can differentiate into various tissues. iPSCs have been successfully differentiated into cartilage and osteogenic lineages driven by TGF-β and BMP and have been widely used in osteochondral tissue engineering (Diekman et al., 2012; Wu et al., 2017; Limraksasin et al., 2020). Therefore, iPSCs have become a powerful cell source for constructing cartilage organoids. Li et al. successfully induced iPSCs to differentiate into cartilage organoids *in vitro* and characterized them, which supported the ability of iPSCs to be the initiating cells of cartilage organoids (Li et al., 2022).

### Mesenchymal stem cells

MSCs, one of the critical members of the stem cell family, were first discovered and isolated, and cultured in the bone marrow. Thus, they are also called bone marrow mesenchymal stem cells (BMSCs) (Haynesworth et al., 1992). In addition to bone marrow, MSCs have been found in other tissues, such as adipose and muscle tissue (Krawczenko and Klimczak, 2022). MSCs have multi-directional differentiation potential *in vivo* or *in vitro*. They can differentiate into bone, cartilage, fat, muscle, and other mesenchymal tissue cells under specific induction conditions (Pittenger et al., 1999) and still have differentiation potential even after continuous subculture and cryopreservation (Bruder et al., 1997). Currently, researchers have used MSCs to generate cartilage organoids. Bian et al. induced cartilage-like tissue formation *in vivo* and *in vitro* by encapsulating MSCs with hyaluronic acid gel, and the local delivery of TGF-β3 can promote the cartilage formation of MSCs (Bian et al., 2011). In addition, Vail et al. compared the microRNA transcriptome level between MSCs cultured cartilage organoids and natural articular cartilage, revealing microRNA’s complex regulatory role in cartilage differentiation (Vail et al., 2022).

### Human periosteum-derived cells

The periosteum is a connective tissue attached to the bone surface and rich in blood vessels. Periosteum-derived cells play an important role in bone development, regeneration, and repair after injury. The periosteum is involved in intramembranous osteogenesis and endochondral osteogenesis (Agata et al., 2007). Studies have shown that periosteal cells and MSCs are derived from the same embryonic mesenchymal lineage. However, compared with MSCs, periosteal cells have higher regenerative capacity, which may be attributed to the skeletal stem cells enriched in the periosteum (Duchamp de Lageneste et al., 2018). In another study, cells isolated from the periosteum of the femoral neck of arthritis patients were cultured and compared with the bone marrow stromal cells (Chang et al., 2014). The hPDCs showed significant similarities in proliferation rate, morphology, and surface receptor expression with bone marrow stromal cells cultured under the same *in vitro* conditions (Chang et al., 2014). Thus, cells isolated from the periosteum are considered a potential source of autologous stem cells. The discovery of pluripotency of periosteum-derived cells makes it possible for them to be used in tissue engineering research. Cartilage microtissues (soft callus organoids) formed by periosteum-derived cells can form bone organs *in vivo* and have the ability to repair bone defects (Nilsson Hall et al., 2020). In addition, callus organoids formed by hPDCs and cartilage microtissues constructed by iPSCs were combined and sequentially differentiated into bone and cartilage *in vivo* (Hall et al., 2021).

## Construction of the culture environment of cartilage organoids

The growth and development of tissues and organs require specific conditions, and stem cell-derived organoids also need a particular culture environment to induce stem cells’ proliferation and differentiation to self-assemble into organoids. In living organisms, the extracellular matrix (ECM) is synthesized by cells and secreted outside the cell. Its main components are polysaccharides and proteins, such as collagen, elastin, laminin, fibronectin, glycosaminoglycans, and other macromolecules, which are capable of forming a complex meshwork structure (Theocharis et al., 2016; Karamanos et al., 2021). Moreover, the extracellular matrix can release various growth factors, cytokines, and chemokines, which are vital for cell adhesion, growth, spreading, and differentiation, connecting and supporting tissue structure stability and regulating tissue cells’ physiological functions (Theocharis et al., 2016; Karamanos et al., 2021).

It is, therefore, necessary to mimic the properties of ECM in natural tissues to provide a better environment required for organoid culture. Several ECM-derived materials have been used for organoid culture (Kim et al., 2020). Matrigel is the most commonly used matrix. It is a gel-like protein mixture secreted by mouse osteosarcoma cells and contains extracellular matrix protein components, such as collagen, fibronectin, and proteoglycan. It is a natural ECM and can be a suitable scaffold for cells to attach and organize into organoids (Kleinman and Martin, 2005). It has been widely used in the study of early organoids. However, the reproducibility of the experiments is limited by the complexity of the composition of the natural matrix glue and the differences between batches. To this end, researchers have developed decellularized ECM as an alternative to organoid culture, which retains the basic components of the natural ECM and can support organoid culture. The culture of endodermal organoids was achieved by obtaining acellular ECM from porcine small intestinal mucosa/submucosa cells by acellular treatment (Giobbe et al., 2019). Furthermore, Crispim et al. using an acellular biological matrix processed from porcine nucleus pulposus tissue as a medium additive, achieved massive proliferation of chondrocytes and self-assembly into organoids similar to hyaline cartilage (Crispim and Ito, 2021). With the rapid development in the field of biomaterials, more matrix glue substitutes have been developed and used for organoid cultures, such as natural biological materials alginate, hyaluronic acid, chitosan, and other polysaccharides, and protein gelatins (e.g., fibrin and collagen and controllable synthetic hydrogels) (Kaur et al., 2021; Kozlowski et al., 2021). The continuous progress of ECM-derived materials provides more suitable culture conditions for organoid culture, which is more conducive to cell proliferation and differentiation (Greggio et al., 2013). It promotes the continued development of organoid research. In addition, introducing a variety of growth factors such as TGF-β, BMP2, and FGF-2 during the culture process can better promote the proliferation and differentiation of stem cells (Wu et al., 2021). Moreover, the appropriate addition of some signaling regulators will more stably induce stem cells to differentiate toward specific cell lineages (Kawata et al., 2019).

## Cultivation methods of cartilage organoids

Achieving self-assembly of three-dimensional cellular structures is one of the critical parts of organoid culture, and the current methods of organoid culture are mainly scaffold-based and scaffold-free.

Scaffold-based culture methods support cell adhesion and proliferation by using biomaterials with multiple pores and self-assembling them into multi-layered organoid structures along specific spatiotemporal orientations. As mentioned above, matrix glue or decellularized matrix and other hydrogel materials, in addition to serving as a cell culture medium to provide a particular cultural environment, can also act as a temporary scaffold for cell self-organization and to maintain the structural stability of organoids to a certain extent (Qu et al., 2017). Xiahou et al. designed a porous hydrogel capable of regulating stem cell-to-hydrogel matrix and cell-to-cell interactions to incubate cartilage microtissues *in vitro* (Xiahou et al., 2020). Crispim et al. developed a new strategy to construct hyaline cartilage *in vitro* (Crispim and Ito, 2021). After applying an ECM to promote the proliferation of chondrocytes, cells and ECM components self-assemble into organoids, and chondrocytes are distributed in the ECM space (Crispim and Ito, 2021). Cells interact with the scaffold material and can form more stable organoids.

In contrast, scaffold-free culture methods are based on the self-organization of cells, placing cells in suspension cultures, or cultivating organoids by developing spherical intermediate tissues. Yamashita et al. produced scaffold-free hyaline cartilage by culturing PSC cells in scaffold-free suspension cultures (Yamashita et al., 2015). Mizuno et al. used bovine cartilage cells from different regions to culture spherical cartilage organoids and reproduced the regional characteristics of articular cartilage at different longitudinal depths (Mizuno et al., 2016). In addition, O'Connor et al. developed a scaffold-free and bioreactor-free method to expose iPSC cells to cartilage continuously and osteogenic-inducing factors such as TGF-β and BMP, thereby mimicking endochondral osteogenesis conditions to generate osteochondral organoids *in vitro* (O'Connor et al., 2021). Other scaffold-free culture methods have also been used in cartilage organoid research. For example, Irie et al. used a hollow fiber as a cell culture device and then induced chondrocytes to form cylindrical organoids by centrifugation (Irie et al., 2008). In addition, tissue engineering research on the production of cartilage and bone tissue from stem cells using rotating bioreactors or simulated microgravity environments has been extensively developed, and microgravity conditions and their applications are well described in some excellent reviews (Aleshcheva et al., 2016; Mochi et al., 2022). Rotating bioreactors and microgravity environments will provide more efficient methods for culturing scaffold-based or scaffold-free cartilage organoids.

In the scaffold-based organoid culture, by using the assistance of scaffolds and the intervention of artificially regulated growth and differentiation factors, cells can proliferate and differentiate better to form complex organoids. Scaffold-free culture, however, is simple and reproducible, enabling massive cell proliferation to form cell aggregates and rapid organoid production.

## Application of cartilage organoids

Although research in cartilage organoids is still in its infancy (Table 1), cartilage organoids have a wide range of potential applications in biological development, disease modeling, drug screening, and regeneration and repair.

**TABLE 1:** Development status of cartilaginous organoids.

Time	References	Cell sources	Organoid characteristics and applications
2016	Mizuno et al. (2016)	chondrocyte	Spheroid organoids formed by chondrocytes in different longitudinal regions with cartilage extracellular matrix components and gene expression characteristic of longitudinal region dependence
2020	Nilsson Hall et al. (2020)	hPDCs	hPDCs differentiated into chondrocyte microspheres and self-assembled into callus organoids (cartilage intermediate). After subcutaneous implantation in mice, they formed bone microorganisms with bone marrow cavities and were able to bridge bone defect tissue
2021	Tam et al. (2021)	hPSCs	hPSCs were induced to differentiate into chondrocytes and assembled into cartilage organoids under suspension culture. After stimulating pro-inflammatory factors, the matrix turnover of cartilage organoids was accelerated to promote the repair of crucial bone defects
2021	Crispim and Ito (2021)	chondrocyte	Chondrocytes were rapidly expanded in the decellularized matrix and self-assembled into cartilage organoids in a rotating flask, and then assembled with viscoelastic hydrogels to generate hyaline cartilage tissue *in vitro*
2021	O'Connor et al. (2021)	miPSCs	After gradual exposure to growth factors (TGF-β3, BMP-2), miPSC underwent endochondral ossification and formed osteochondral organoids *in vitro*
2022	Abraham et al. (2022)	chondrocyte	Bone and cartilage organoids were generated by organoid culture, and osteochondral dual-tissue organoids were constructed by the co-culture method to establish an arthritis model and drug testing
2022	Li et al. (2022)	MSCs, iPSCs	MSCs and iPSC differentiated to form bone, cartilage, and adipose organoids in the ECM secreted by stem cells, and osteochondral organoids with high ossification centers and cartilaginous shells were obtained by induction of osteogenic and chondrogenic sequences
2023	Abe et al. (2023)	iPSCs	After differentiation, iPSCs were induced to form cartilage organoids by suspension culture and chondrogenic medium. Cartilage organoids were transplanted into cartilage defects to form articular cartilage, and the expression of proteoglycan 4 (PRG4) was increased
2023	Yang et al. (2023)	MSCs	MSCs were induced into cartilage and osteogenesis in custom-made gels and self-assembled into osteochondral organoids *in vitro*, enabling simultaneous cartilage and bone regeneration *in vivo*

hPDCs, human periosteal-derived cells; hPSCs, human pluripotent stem cells; miPSCs, mouse induced pluripotent stem cells; MSCs, mesenchymal stem cells; iPSCs, induced pluripotent stem cells. TGF-β3, transforming growth factor β3; BMP-2, bone morphogenetic protein 2.

### Building disease models

Organoid structures are generated from pluripotent stem cells or adult cells by induced differentiation and through their self-organizing properties. The organoid composition and structure are similar to the originating tissue and reproduce some of the physiological functions of similar organs. Therefore, organoids are good models for simulating development and studying diseases (Fatehullah et al., 2016). Joint diseases such as OA have become common worldwide, causing pain and disability (GBD, 2017 Disease and Injury Incidence and Prevalence Collaborators et al., 2018). Currently, animal experiments and two-dimensional cell experiments are still the mainstream models for preclinical OA research. However, simple monolayer cell cultures lack intercellular interactions and cannot simulate the complex physiological situation between tissues; although animal models can partially compensate for cell cultures, they are also limited by structural and physiological differences between species and substantial economic costs (Bjornson-Hooper et al., 2022). Although other *in vitro* research models, such as tissue explant models, bioreactor models, and organ-on-chips, have also been developed for OA research (Singh et al., 2021). These *in vitro* models can increase the understanding of different cell-cell interactions and imitate and recapitulate some human tissues and organs’ complex physiological microenvironments and functions, which may reduce the need for animal experiments to a certain extent (Singh et al., 2021). However, these models are still relatively new and require continuous optimization and innovation. Osteochondral organoids with similar structures and physiological functions will be an ideal model for the study of bone and joint development and diseases in the future. For example, OA is a complex disease involving the whole joint, and cartilage and subchondral bone play a vital role in the initiation and development of OA. Through organoid culture, *in vitro* disease models with more structural components can be constructed. For example, Abraham et al. constructed a “mini-joint” composite bone and cartilage model with cells isolated from bone and cartilage tissue (Abraham et al., 2022). Furthermore, the constructed osteochondrosis organoids were treated with interleukin-1β [a proinflammatory factor that is closely related to the occurrence and development of osteoarthritis (Kapoor et al., 2011)] to establish a joint inflammation model (Abraham et al., 2022). In addition, this multi-level model containing bone and cartilage can simulate the excessive area in the joint, thus providing a better *in vitro* model for studying the role of subchondral bone in the pathogenesis of OA (Abraham et al., 2022).

### Drug screening and testing

Most drugs require lengthy research and *in vitro* and *in vivo* testing before clinical approval. Particularly, the limitations of animal and simple cellular models lead to the need for the representativeness of drug testing and screening in animal and cellular experiments, which only partially reflects the true efficacy and toxic effects of drugs in humans. Translating drug development from traditional animal and cell-based experiments to the clinic has yet to be very seamless. The construction of organoids to simulate the pathophysiological environment of human diseases will provide new ideas for drug screening and testing (Rossi et al., 2018). As described above, joint inflammation models were constructed by cartilage organoid cultures and used to test the protective effect of the drug A2AR agonist on inflammation (Abraham et al., 2022) [A2AR is an adenosine receptor agonist, which targets adenosine receptors that play an essential role in articular cartilage homeostasis and arthritis development (Corciulo et al., 2017)]. Cartilage organoids are supported as a novel *in vitro* drug screening and testing models. Furthermore, using engineering techniques to rapidly and massively produce cartilage organoids for high-throughput drug screening provides a more advantageous research strategy for drug development of OA.

### Regeneration and restoration engineering

Cartilage destruction is the main pathological change of OA, but due to the unique structural and physiological properties of articular cartilage, cartilage repair and reconstruction becomes a great challenge. Currently, the clinical intervention methods for cartilage defects mainly include microfracture and autologous or allogeneic chondrocyte transplantation. Still, due to the limited donor tissue and the poor integration of the graft with the surrounding tissue, it takes work to achieve good expected results (Richter et al., 2016; Riboh et al., 2017). Interventions based on regenerative repairs, such as cell therapy to promote cartilage defect repair by injecting stem cells directly into the joint cavity (Zelinka et al., 2022), have shown promising results in preclinical and clinical studies and are progressing toward clinical translation but still face many problems. Integrating implants into native cartilage tissue is a crucial step in cartilage repair and one of the critical challenges in cartilage regeneration and repair research. A recent study demonstrated the integration between organoid implants and native cartilage tissue from OA patients (Kleuskens et al., 2022). By implanting cartilage organoids cultured *in vitro* into explants obtained from the human tibial plateau, the integration and interaction of the implanted cartilage organoids with the surrounding native cartilage were observed (Kleuskens et al., 2022). This experiment demonstrates a promising implantation strategy for cartilage organoids as grafts for cartilage defect repair. In another study, cartilage organoids derived from allogeneic induced pluripotent stem cells were transplanted into articular cartilage defects of non-primates to achieve cartilage tissue repair at the defect site (Abe et al., 2023). Histological staining also confirmed that the repaired tissue was hyaline cartilage rather than fibrocartilage, and the fixed tissue remained active for an extended period (Abe et al., 2023). This kind of cartilage tissue repair in the form of hyaline cartilage will provide a new option for treating articular cartilage defects. More interestingly, osteochondral organoids with dual bone and cartilage tissue structures have achieved layered cartilage and subchondral bone regeneration *in vivo* (Yang et al., 2023).

**FIGURE 3:**
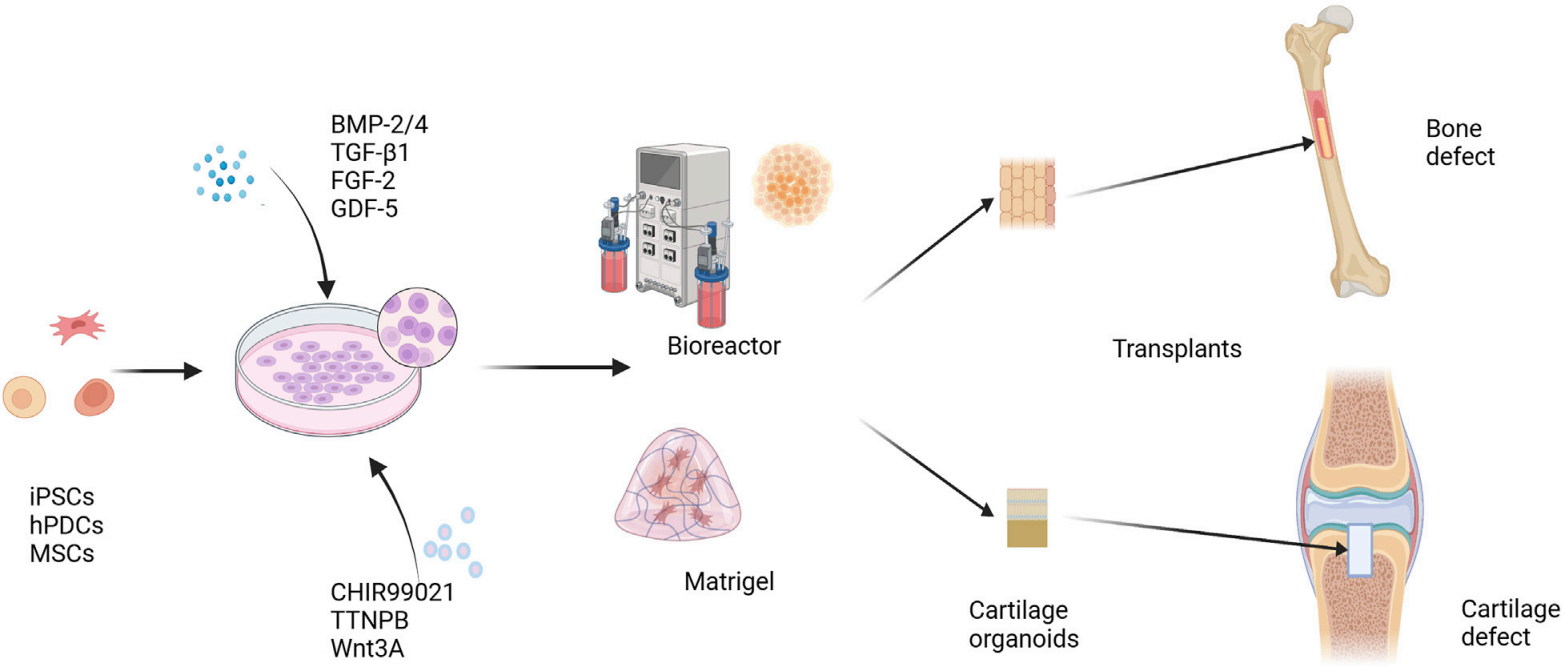
Cartilage organoids and regeneration repair. iPSCs, hPDCs, and MSCs are induced to differentiate under the influence of growth and differentiation factors, including BMP-2/4, TGF-β1, FGF-2, and GDF-5. A variety of cell signals are involved in regulating the differentiation and maturation of chondrocytes. Small molecule compounds such as CHIR99021, TTNPB, and Wnt3A can promote the differentiation and maturation of stem cells into chondrocytes more stably. Cells spread and grow on Matrigel scaffolds or in a scaffold-free environment, and form cartilage organoids. Using cartilage organoids as grafts can be used to repair cartilage defects and bone defects. BMP-2, bone morphogenetic protein 2; TGF-β1, transforming growth factor β1; FGF-2, fibroblast growth factor 2; GDF-5, growth differentiation factor 5; CHIR99021, a glycogen synthase kinase 3 inhibitor; TTNPB, a retinoic acid receptor agonist. iPSCs, induced pluripotent stem cells; hPDCs, human periosteal-derived cells; MSCs, mesenchymal stem cells.

The above studies demonstrate that cartilage organoids have substantial application potential in OA *in vitro* modeling and drug screening. Cartilage organoids have demonstrated impressive therapeutic effects in the repair of articular cartilage defects. In the future, developing new OA disease models and new treatment strategies using cartilage organoids will further promote the understanding and treatment of OA disease. In fact, cartilage organoids have more than just applications in skeletal diseases. Studies by Nilsson Hall et al. (2020) and Tam et al. (2021) have shown that after constructing callus organoids (a cartilage intermediate) and priming them by targeting hypertrophy-promoting and pro-inflammatory mediators, callus organoids can bridge bone tissue around critical size bone defects and promote bone defect repair. In addition, combining the 3D bioprinting technology of DLP (digital light processing) with a microsphere-based culture system can realize the engineering of bone callus tissue organoids and achieve rapid bone regeneration and repair (Xie et al., 2022) (Figure 3).

## Summary and prospects

Although the research on cartilage organoids is still in its infancy, from the current findings, it is clear that cartilage organoids show great potential in tissue engineering. Cartilage organoids have shown positive research value in biological development, disease modeling, drug screening, regeneration, and repair. However, the current research on cartilage organoids still faces many challenges.

Firstly, cartilage organoids have the original tissue’s basic structure and some physiological functions. However, due to tissue cells’ diversity and physiological complexity *in vivo* (tissue cells interact with cells through complex signal factors to exert different physiological functions), cartilage organoids cannot fully reproduce the physiological environment *in vivo*. More studies are needed to address these shortcomings. Secondly, the maturity of cells is also an important issue, which may limit the application of organoids in biological development models (Akiva et al., 2021; Steinhart et al., 2023). The differentiation and maturation of cells require complex biological information regulation and nutritional support, which may be improved by adding appropriate signaling factors during culture and developing better systems to promote the distribution of nutrients. Rotary bioreactors and microfluidic culture systems have been shown to facilitate nutrient and oxygen diffusion (Jeong et al., 2023), but more research explorations are needed. Furthermore, because intact organs consist of multiple types of cells and interact with each other, how to construct cartilaginous organoids with multiple lineages is the direction of future research, and the top-down co-culture model provides some reference value but still needs to be improved. Finally, as the load-bearing tissue of the body, the biomechanical properties of cartilage organoids still need further consideration. It is expected to study cartilage organoids that exhibit normal mechanical stress in organisms to be better used in the research and treatment of bone diseases.

In addition, many problems need to be paid attention to in applying cartilage organoids. For example, in treating bone and cartilage defects, cartilage organoids have been shown to integrate the surrounding tissue better. However, further studies are still needed to maintain cellular phenotype and biological properties after transplantation. 3D printing techniques and tissue engineering techniques are dedicated to the construction of vascularized bone tissue constructs, and it has been proved that the formation of vascular networks contributes to the osteogenic differentiation of MSCs (Hann et al., 2021). Therefore, using cartilage organoids as bio-ink combined with a 3D printing technique to construct callus organoids with vascular components may better promote the regeneration and repair of bone defects.

Although there are still many challenges in the research and application of cartilage organoids, it has broad prospects in bone and joint development and disease simulation, drug screening, and regenerative medicine. The continuous development of techniques, such as biomaterials science, 3D bioprinting technique, organ-on-a-chip technique, and new gene editing techniques, will undoubtedly help organoids further exert their potential.
